# Placenta-derived exosomes continuously increase in maternal circulation over the first trimester of pregnancy

**DOI:** 10.1186/1479-5876-12-204

**Published:** 2014-08-08

**Authors:** Suchismita Sarker, Katherin Scholz-Romero, Alejandra Perez, Sebastian E Illanes, Murray D Mitchell, Gregory E Rice, Carlos Salomon

**Affiliations:** UQ Centre for Clinical Research, Centre for Clinical Diagnostics, Royal Brisbane and Women’s Hospital, University of Queensland, Building 71/918, Herston, QLD 4029 Queensland Australia; Department of Obstetrics and Gynaecology, Faculty of Medicine, Universidad de los Andes, Santiago, Chile; Department of Obstetrics and Gynaecology, Perinatal unit, Clinica Dávila, Santiago, Chile

**Keywords:** Exosomes, Pregnancy, Placenta, Fetal-maternal exchange

## Abstract

**Background:**

Human placenta releases specific nanovesicles (*i.e.* exosomes) into the maternal circulation during pregnancy, however, the presence of placenta-derived exosomes in maternal blood during early pregnancy remains to be established. The aim of this study was to characterise gestational age related changes in the concentration of placenta-derived exosomes during the first trimester of pregnancy (*i.e.* from 6 to 12 weeks) in plasma from women with normal pregnancies.

**Methods:**

A time-series experimental design was used to establish pregnancy-associated changes in maternal plasma exosome concentrations during the first trimester. A series of plasma were collected from normal healthy women (10 patients) at 6, 7, 8, 9, 10, 11 and 12 weeks of gestation (n = 70). We measured the stability of these vesicles by quantifying and observing their protein and miRNA contents after the freeze/thawing processes. Exosomes were isolated by differential and buoyant density centrifugation using a sucrose continuous gradient and characterised by their size distribution and morphology using the nanoparticles tracking analysis (NTA; Nanosight™) and electron microscopy (EM), respectively. The total number of exosomes and placenta-derived exosomes were determined by quantifying the immunoreactive exosomal marker, CD63 and a placenta-specific marker (Placental Alkaline Phosphatase PLAP).

**Results:**

These nanoparticles are extraordinarily stable. There is no significant decline in their yield with the freeze/thawing processes or change in their EM morphology. NTA identified the presence of 50–150 nm spherical vesicles in maternal plasma as early as 6 weeks of pregnancy. The number of exosomes in maternal circulation increased significantly (ANOVA, *p* = 0.002) with the progression of pregnancy (from 6 to 12 weeks). The concentration of placenta-derived exosomes in maternal plasma (*i.e.* PLAP^+^) increased progressively with gestational age, from 6 weeks 70.6 ± 5.7 pg/ml to 12 weeks 117.5 ± 13.4 pg/ml. Regression analysis showed that weeks is a factor that explains for >70% of the observed variation in plasma exosomal PLAP concentration while the total exosome number only explains 20%.

**Conclusions:**

During normal healthy pregnancy, the number of exosomes present in the maternal plasma increased significantly with gestational age across the first trimester of pregnancy. This study is a baseline that provides an ideal starting point for developing early detection method for women who subsequently develop pregnancy complications, clinically detected during the second trimester. Early detection of women at risk of pregnancy complications would provide an opportunity to develop and evaluate appropriate intervention strategies to limit acute adverse sequel.

## Background

The placenta plays a pivotal role in mediating maternal adaptation to pregnancy as well as regulating fetal growth and development. Pregnancy-induced changes are affected by the release of soluble autacoids as early as 6 to 8 weeks of gestation [1, 2] and the invasion of placental cells into the maternal tissues to modify maternal immune, cardiovascular and metabolic activities. Recently, we and others [3–7] have identified an additional pathway by which the placenta communicates with the maternal system to induce changes during pregnancy-placental exosomal signalling.

Exosomes are bilipid membrane-bound nanovesicles (50–120 nm diameter) that are actively released (via exocytosis) from cells into the extracellular space and body fluids under physiological and pathophysiological conditions [8]. Their molecular cargo of proteins, microRNAs, mRNAs and lipids appear to be selectively packaged by the late endosomal system to regulate the phenotype of target cells [3, 4, 6]. Recent studies have highlighted the putative utility of tissue-specific nanovesicles (*e.g.* exosomes) in the diagnosis of disease onset and treatment monitoring [4, 9, 10].

Previously, we have established that placental cells release exosomes in response to changes in the extracellular milieu (including oxygen tension and glucose concentration) and that placental cell-derived exosomes regulate target cell migration and invasion [3, 4]. In addition, we have identified placental-derived exosomes in maternal blood and reported that the concentration of placental exosomes in the maternal blood increases during normal, healthy pregnancy [7]. During early placentation, the cytotrophoblast cells form a highly invasive extravillous trophoblast that can migrate into the decidua and invade the first third of the myometrium, inducing remodelling of spiral arterioles to produce low-resistance vascular system, essential for fetal development [11]. The relative reduction of utero-placental flow caused by abnormal placentation triggers the development of placental originated diseases such as preeclampsia. Available data suggest that the concentrations of placental-derived exosomes in the maternal blood could be a potential marker of abnormal placentation [12, 13].

Early detection of disease risk and onset is the first step in implementing efficacious treatment and improving patient outcome. To date, the concentration profile of placenta-derived exosomes in the maternal blood during first trimester has not been established. Until this profile is defined, the utility of placental exosomes as an early biomarker for placental dysfunction will remain equivocal. In this study, therefore, a time-series experimental design was used to test the hypothesis that the concentration of placental exosomes in the maternal plasma of normal healthy women changes during the early pregnancy state (*i.e*. 6–12 weeks).

## Methods

### Patient selection and sample collection

A time-series experimental design was used to establish the variation in plasma exosome characteristics during normal pregnancy. All experimental procedures were conducted within an ISO17025 accredited (National Association of Testing Authorities, Australia) research facility. All data were recorded within a 21 CERF part 11 compliant electronic laboratory notebook (Iris note, Redwood City, CA, USA). Plasma samples were collected from 10 women during their first trimester of pregnancy. All patients were enrolled with informed consent and underwent routine obstetrical care at the Hospital Parroquial de San Bernardo (Santiago, Chile). Estimation of gestational age was made based on the first day of their last menstrual period and confirmed by transvaginal ultrasound at the recruitment (*i.e.* 6 weeks). Each patient, gave consent to have weekly blood sample collection between 6 and 12 weeks of gestation (n = 70, 10 patients with weekly blood collection at 6, 7, 8, 9, 10, 11 and 12 weeks of pregnancy). The protocol of the study was approved by the Institutional Review Board of the Universidad de los Andes (Santiago, Chile). Obstetrical history and physical findings were recorded regarding previous spontaneous abortions, course of previous pregnancies, hypertension, gestational diabetes and preeclampsia. Peripheral venous blood samples were collected in EDTA treated tubes (BD Vacutainer® Plus plastic plasma tube) from which plasma samples were obtained by centrifugation at 2000 × g at 4°C for 10 min. The plasma samples were stored in aliquots at −80°C until analysed (not more than three months).

### Exosome isolation

Exosomes were isolated as previously described [3, 4, 7, 14]. Briefly, plasma from each patient was utilised to isolate exosomes. Plasma (2.5 ml) was diluted with equal volume of PBS (pH 7.4) and exosomes were isolated through differential centrifugation, microfiltration and buoyant density ultracentrifugation. Centrifugation was initially performed at 2,000 × g at 4°C for 30 min (Thermo Fisher Scientific Ins., Asheville, NC, USA, Sorvall®, high speed microcentrifuge, fixed rotor angle: 90°) followed by 12,000 × g at 4°C for 45 min to sediment cell nuclei, mitochondria and debris. The supernatant fluid (~5 ml) was transferred to an ultracentrifuge tube (Ultracrimp tubes, Thermo Fisher Scientific Ins., Asheville, NC, USA) and was centrifuged at 200,000 × g at 4°C for 2 h (Thermo Fisher Scientific Ins., Asheville, NC, USA, Sorvall®, T-8100, fixed angle ultracentrifuge rotor). The pellet was suspended in PBS (5 ml) and filtered through a 0.22 μm filter (SteritopTM, Millipore, Billerica, MA, USA). The filtrate was centrifuged at 200,000 × g at 4°C for 70 min (Thermo Fisher Scientific Ins., Asheville, NC, USA, Sorvall®, T-8100, fixed angle ultracentrifuge rotor) and the pellet resuspended in 2.5 M sucrose (4 ml).

### Purification of exosomes using a continuous sucrose gradient

The resuspended 200,000 g pellet in 2.5 M sucrose was added at the bottom of an ultracentrifuge tube. A continuous sucrose gradient (26 ml; 0.25-2.5 M) was made above 4 ml of exosome suspension using a Hoefer SG30 gradient maker (GE Healthcare, NSW, Australia) and centrifuged at 110,000 g for 20 h (Sorvall, SureSpin™ 630/360, Swinging-Bucket ultracentrifuge rotor). Fractions (10 in total, 3 ml each) were collected automatically using a Pulse-Free Flow Peristaltic Pump with a flow rate range of 3 ml per min (GILSON Miniplus® model 3) and the Fraction Collector (GILSON FC 203B model). The density of each fraction was determined using the refraction index with OPTi digital refractometer (Bellingham + Stanley Inc., Lawrenceville, GA, USA). The coefficient of variation (CV) was less than 8% for the density of each fraction. Fractions (3 ml each) were diluted in PBS (60 ml) and then centrifuged at 200,000 × g for 70 min. The 200,000 g pellet was resuspended in 50 μl PBS and stored at −80°C. Exosomal protein concentrations were determined by a colorimetric assay (DC™ Protein Assay, Bio-Rad Laboratories, Hercules, CA, USA) [4].

### Identification of nanoparticles by nanoparticle tracking analysis (NTA)

NTA measurements were performed using a NanoSight NS500 instrument (NanoSight NTA 2.3 Nanoparticle Tracking and Analysis Release Version Build 0033) following the manufacturer’s instructions. The NanoSight NS500 instrument measured the rate of Brownian motion of nanoparticles in a light scattering system that provides a reproducible platform for specific and general nanoparticle characterization (NanoSight Ltd., Amesbury, United Kingdom). Samples were processed in duplicates and diluted with PBS over a range of concentrations to obtain between 10 and 100 particles per image (optimal ~50 particles x image) before analysing with NTA system. The samples were mixed before introducting into the chamber (temperature: 25°C and viscosity: 0.89 cP) and the camera level set to obtain image that has sufficient contrast to clearly identify particles while minimizing background noise a video recording (camera level: 10 and capture duration: 60 s). The captured videos (2 videos per sample) were then processed and analysed. A combination of high shutter speed (450) and gain (250) followed by manual focusing enabled optimum visualization of a maximum number of vesicles. We included a minimum of 200 tracks completed per video in duplicates. NTA post acquisition settings were optimized and kept constant between samples (Frames Processed: 1496 of 1496, Frames per Second: 30, camera shutter: 20 ms; Calibration: 139 nm/pixel, Blur: 3×3; Detection Threshold: 10; Min Track Length: Auto; Min Expected Size: Auto), and each video was then analyzed to give the mean, mode, and median particles size together with an estimate of the number of particles. An Excel spreadsheet (Microsoft Corp., Redmond, Washington) was also automatically generated, showing the concentration at each particle size.

### Transmission electron microscopy (TEM)

For the TEM analysis, exosome pellets (as described above, 30 μg protein) were fixed in 3% (w/v) glutaraldehyde and 2% paraformaldehyde in cacodylate buffer, pH 7.3. Exosome samples were then applied to a continuous carbon grid and negatively stained with 2% uranyl acetate. The samples were examined in an FEI Tecnai 12 transmission electron microscope (FEI™, Hillsboro, Oregon, USA) in the Central Analytical Research Facility, Institute for Future Environments, Queensland University of Technology (QUT) (see Acknowledgements).

### Quantification of placental cell-derived exosome

The concentration of exosomes in maternal circulation was expressed as the total immunoreactive exosomal CD63 (ExoELISA™, System Biosciences, Mountain View, CA). Briefly, 10 μg of exosomal protein was immobilised in 96-well microtiter plates and incubated overnight (binding step). Plates were washed three times for 5 min using a wash buffer solution and then incubated with exosome specific primary antibody (CD63) at room temperature (RT) for 1 h under agitation. Plates were washed and incubated with secondary antibody (1:5000) at RT 1 h under agitation. Plates were washed and incubated with Super-sensitive TMB ELISA substrate at RT for 45 min under agitation. The reaction was terminated using Stop Buffer solution. Absorbance was measured at 450 nm. The number of exosomes/ml, (ExoELISA™ kit) was obtained using an exosomal CD63 standard curve calibrated against nanoparticle tracking data (*i.e.* number of exosomes, NanoSight™).

For placental cell-derived exosomes, the concentration of exosomal PLAP was quantified using a commercial ELISA kit (MYBioSource MBS701995, San Diego, CA, USA) according to manufacturer’s instructions (detection range: 84–2000 pg/ml; sensitivity: 34 pg/ml; intra-assay precision within an assay: CV% < 10%; inter-assay between assays: CV% < 15%) Briefly, 10 μg of exosomal protein was added to each well of a 96-well microtitre plate and incubated at 37°C for 30 min. Plates were washed three times while shaking for 20 s and 50 μl of HRP-conjugate was added to each well and incubated at 37°C for 20 min. Plates were washed and incubated with 50 μl of substrate A and 50 μl of substrate B at 37°C for 15 min. The incubation was terminated using 50 μl of stop solution at RT for 2 min under agitation. Absorbance was measured at 450 nm. Exosomal PLAP was expressed as pg PLAP /ml plasma.

### Stability of the exosomal quantification

To determine the stability of the exosomes during freeze-thaw cycles, fresh plasma (5.0 ml) from healthy women were obtained and divided into two 2.5 ml samples (A and B). Exosomes were immediately isolated from the first aliquot (A: fresh plasma) by differential and buoyant density centrifugation and then characterised by the number of exosome particles using an ELISA kit (ExoELISA™, System Biosciences, Mountain View, CA), morphologically by electron microscope, microRNA content by real time PCR and protein profiling by mass-spectrometry. Sample B plasma was stored at −80°C for 2 months (B: frozen plasma), prior to exosome isolation and characterisation. *miRNA isolation*: miRNA were isolated from exosome particles as we have previously described [14]. Ambion mirVana PARIS Kit (Invitrogen, USA) was used to extract exosomal total RNA from fresh and frozen plasma by following the manufacturer’s procedure. Exosomes were first lysed by adding cell disruption buffer and vortexed or pipetted vigorously. Denaturing solution was added to samples and incubated on ice for 5 min. The first two steps stabilize RNA and inactivate RNases. The lysate is then subjected to Acid-Phenol:Chloroform extraction by adding Acid-Phenol:Chloroform, vortexed and centrifuged at 10,000 × g for 5 min. Recovery of the aqueous phase obtains semi-pure RNA samples, removing most of the other cellular components. 100% ethanol was mixed and passed through a filter cartilage. The filter was washed three times and the RNA was eluted with nuclease-free water. *Real-time PCR:* Reverse transcription was performed using the miScript Reverse Transcription Kit (QIAGEN, Valencia, CA, USA) in a total volume of 20 μl. cDNA was synthesised from the maximum volume of exosomal RNA (12 μl) using the BIO-RAD T100™ Thermal Cycler (USA) running for 60 min at 37°C, 5 min for 95°C and 60 min for 37°C. As the control, RNase-free water was added as the RNA template. Real-time PCR was performed with miScript SYBR Green Kit (QIAGEN, Valencia, CA, USA). Forward primers (miScript primer assays, QIAGEN, Valencia, CA, USA) designed to detect the housekeeping gene, human RNU6-2 (RNU6B) was used. The reactions were performed in triplicate using the BIO-RAD iQ™5 Multicolor Real-Time PCR Detection System (USA) with the following conditions: 94°C for 3 min, 35 amplification cycles of 94°C for 45 s, 55°C for 30 s and 72°C for 30 s, 72°C for 10 min, 12°C for ∞ min. *Proteomic analysis of exosomes by mass spectrometry (MS):* We utilised a Liquid Chromatography (LC) and Mass Spectrometry (MS) LC/MS/MS instrumentation available within the University of Queensland Centre for Clinical Research (5500qTRAP and 5600 Triple TOF) to undertake in depth quantitative proteomic analysis of the exosome samples (isolated from fresh and frozen plasma) to determine the proteome of exosomes as we have previously published [4]. Briefly, exosomes were adjusted to 8 M urea in 50 mM ammonium bicarbonate, pH 8.5, and reduced with tris (2-carboxyethyl) phosphine (5 mM) at room temperature for 1 h. Proteins were then alkylated in 10 mM IAA for 1 h in the dark. The sample was diluted 1:10 with 50 mM ammonium bicarbonate and then digested with trypsin (20 μg) at 37°C for 18 h. The samples were dried by centrifugal evaporation to remove the acetonitrile and then redissolved in Solvent A. The digested protein samples were analysed using a 5600 Triple TOF mass spectrometer (ABSciex) to obtain initial high mass accuracy survey MS/MS data, identifing the peptides present in the samples. The in depth proteomic analysis was performed using the Information Dependent Acquisition (IDA) experiments on the 5600 Triple TOF MS and utilized an enhanced MS survey scan (m/z350–1500) followed by 50 data-dependent product ion scans of the 50 most intense precursor ions. The MS data was analysed with the Markerview software package using Principal Components Analysis (PCA) or PCA-Discriminate Analysis (PCA-DA) which compares data across multiple samples, groupings the data sets, and graphically showing the groups in a Scores plot. The Loadings plot provides valuable insight into variables that lead to sample clustering and illustrates which biomarkers are up- or down-regulated. All mass spectra were analysed using the Mascot and Protein Pilot search engines against the Swissprot-swissprot database with the species set as human (scores greater than 30). False discovery rate (FDR) was estimated using a reversed sequence database. Finally, proteins identified were submitted to bioinformatic pathway analysis (Ingenuity Pathway Analysis [IPA]; Ingenuity Systems, Mountain View, CA; http://www.ingenuity.com).

### Statistical analysis

Data are presented as mean ± SEM, with n = 10 different patients per group (*i.e.* 6, 7, 8, 9, 10, 11, 12 weeks). The effect of gestational age on number of exosome particles and placental-derived exosomes were assessed by two-way ANOVA, with variance partitioned between gestational age and subject. Statistical difference between group means was assessed by Dunn’s test to compare each treatment to the control group where the data distribution approximates normality and by Mann–Whitney U-test for distribution independent data analysis. Two group means were statistically assessed by Student’s t-test. Statistical significance was defined as p < 0.05.

## Results

### Exosome characterisation

**Figure 1:**
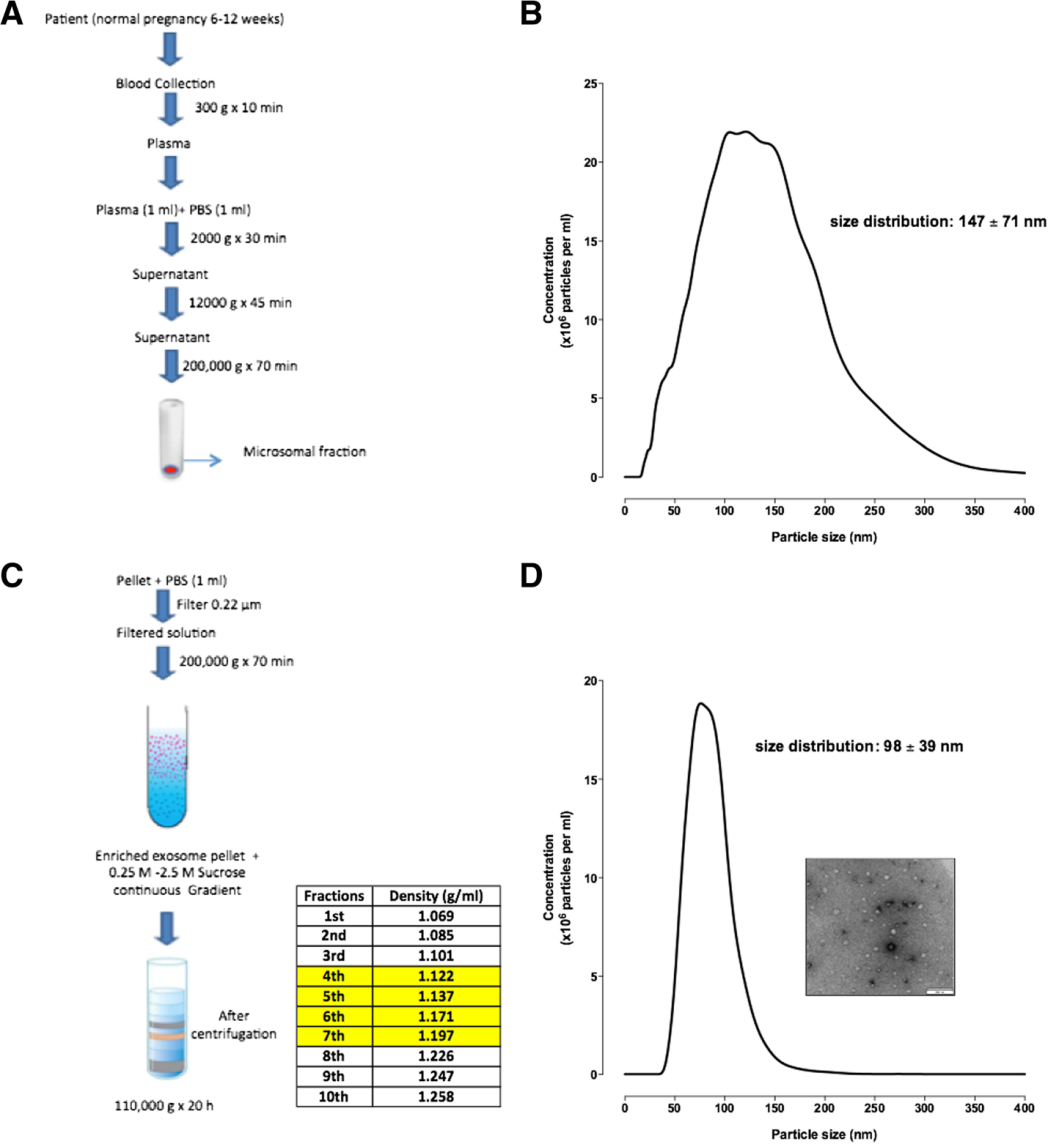
**Characterisation of exosome from maternal circulation.** Exosome were isolated from women uncomplicated pregnancies during first trimester by differential and buoyant density centrifugation (see Methods). **(A)** Flow chart for the exosome purification procedure based on differential ultracentrifugation. **(B)** Representative particles size distribution of microsomal fraction. **(C)** Flow chart for the exosome purification procedure based on sucrose continuous gradient (exosome enriched fractions in yellow 4–7). **(D)** Representative particles size distribution of enriched exosomal fractions (fraction 4–7 were mixed). Insert: Representative electron micrograph exosome fractions (pooled enriched exosome population from fractions 4 to 7), Scale bar 200 nm.

Maternal plasma exosomes isolated by differential and sucrose density gradient centrifugation were characterised by a buoyant density of 1.122 to 1.197 g/ml (fractions 4 to 7) (Figure 1A-D). Nanoparticle tracking analysis showed a particle size distribution of 200,000 × g pellet (Figure 1A) ranging from 30 to 300 nm in diameter corresponding to microsomal fraction (including exosomes particles) with an average of 147 ± 71 nm (mean ± SD) (Figure 1B). After the sucrose continuous gradient, we mixed the enriched exosomal fractions (1.122 to 1.197 g/ml) (Figure 1C) and obtained a particle size distribution ranged from 50 to 140 nm in diameter, with an average of 98 ± 39 nm (mean ± SD) (Figure 1D). Electron microscopy revealed the presence of spherical vesicles, with a typical cup-shape and diameters ranging from 30 to 120 nm (Figure 1D, insert).

**Figure 2:**
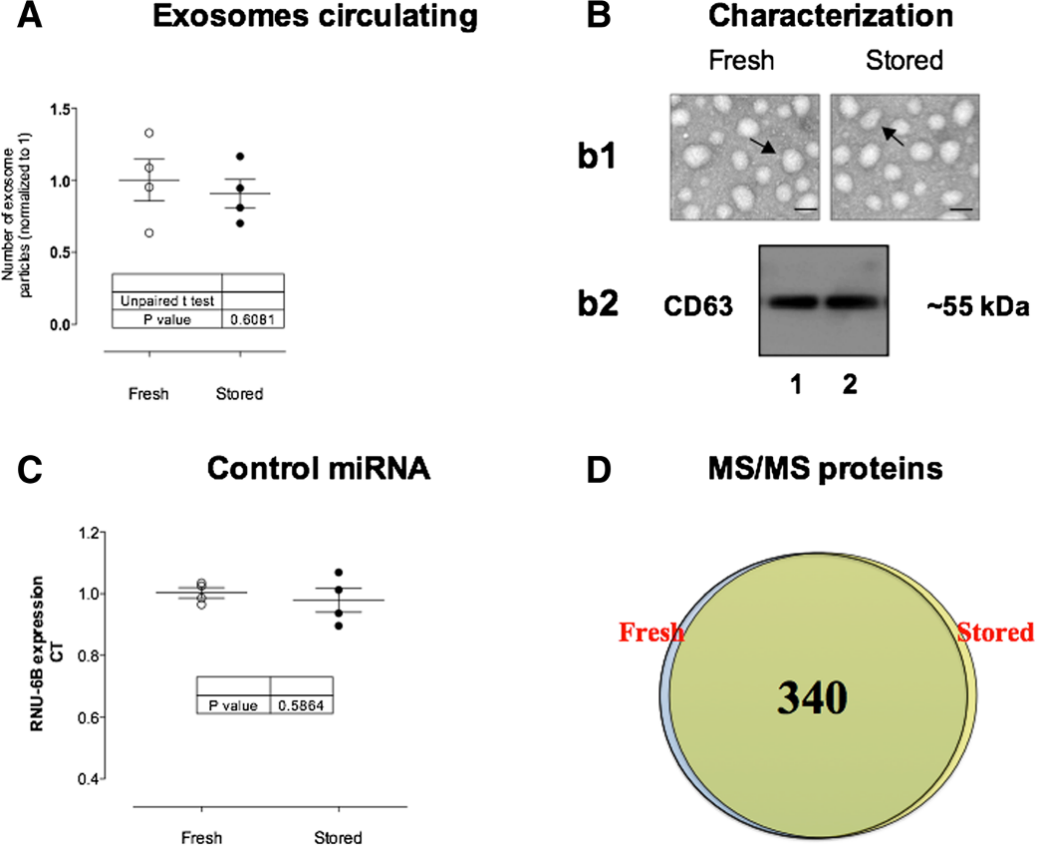
**Characteristics of exosomes isolated from plasma immediately after phlebotomy (○) and after 30 days stored at −80°C (●). (A)** Number of exosome particles. **(B)** Exosomes characterization. *b1*: electron microscope (scale bar 100 nm) and *b2*: Western blot for CD63 (exosomal marker); lane 1: Fresh and lane 2: stored. **(C)** Expression of miRNA RNU6B in exosomes. **(D)** Venn diagram of proteins identified in fresh and stored exosomes.

The stability of exosomes after a freeze and thaw cycle was evaluated using fresh and frozen plasma. No significant difference was observed using fresh or frozen plasma in exosome quantification, exosomal marker expression, microRNA expression or protein content (Figure 2A-D, Table 1).

**Table 1:** **Common proteins identified in exosomes isolated from fresh plasma and after freeze/thawing cycles**

Protein ID	Symbol	Entrez gene name	Location	Type(s)
A2MG_HUMAN	A2M	alpha-2-macroglobulin	Extracellular Space	transporter
A2ML1_HUMAN	A2ML1	alpha-2-macroglobulin-like 1	Cytoplasm	other
ACACA_HUMAN	ACACA	acetyl-CoA carboxylase alpha	Cytoplasm	enzyme
ACTN3_HUMAN	ACTN3	actinin, alpha 3	Plasma Membrane	other
ADAL_HUMAN	ADAL	adenosine deaminase-like	Cytoplasm	enzyme
ATS16_HUMAN	ADAMTS16	ADAM metallopeptidase with thrombospondin type 1 motif, 16	Extracellular Space	other
ATS9_HUMAN	ADAMTS9	ADAM metallopeptidase with thrombospondin type 1 motif, 9	Extracellular Space	peptidase
DSRAD_HUMAN	ADAR	adenosine deaminase, RNA-specific	Nucleus	enzyme
ADCY7_HUMAN	ADCY7	adenylate cyclase 7	Plasma Membrane	enzyme
KFA_HUMAN	AFMID	arylformamidase	Nucleus	enzyme
ANGT_HUMAN	AGT	angiotensinogen (serpin peptidase inhibitor, clade A, member 8)	Extracellular Space	growth factor
ALBU_HUMAN	ALB	albumin	Extracellular Space	transporter
AMZ1_HUMAN	AMZ1	archaelysin family metallopeptidase 1	Other	peptidase
ANK2_HUMAN	ANK2	ankyrin 2, neuronal	Plasma Membrane	other
ANKAR_HUMAN	ANKAR	ankyrin and armadillo repeat containing	Nucleus	transcription regulator
AKD1B_HUMAN	ANKDD1B	ankyrin repeat and death domain containing 1B	Other	other
ANKL1_HUMAN	ANKLE1	ankyrin repeat and LEM domain containing 1	Other	other
ANR12_HUMAN	ANKRD12	ankyrin repeat domain 12	Nucleus	other
ANR26_HUMAN	ANKRD26	ankyrin repeat domain 26	Nucleus	transcription regulator
ANKUB_HUMAN	ANKUB1	ankyrin repeat and ubiquitin domain containing 1	Other	other
APOA1_HUMAN	APOA1	apolipoprotein A-I	Extracellular Space	transporter
APOB_HUMAN	APOB	apolipoprotein B	Extracellular Space	transporter
APOL1_HUMAN	APOL1	apolipoprotein L, 1	Extracellular Space	transporter
APOP1_HUMAN	APOPT1	apoptogenic 1, mitochondrial	Cytoplasm	other
DP13B_HUMAN	APPL2	adaptor protein, phosphotyrosine interaction, PH domain and leucine zipper containing 2	Cytoplasm	other
RHG15_HUMAN	ARHGAP15	Rho GTPase activating protein 15	Cytoplasm	other
RHG08_HUMAN	ARHGAP8/PRR5-ARHGAP8	Rho GTPase activating protein 8	Cytoplasm	other
ARHGB_HUMAN	ARHGEF11	Rho guanine nucleotide exchange factor (GEF) 11	Cytoplasm	other
ASPM_HUMAN	ASPM	asp (abnormal spindle) homolog, microcephaly associated (Drosophila)	Nucleus	other
ATG2B_HUMAN	ATG2B	autophagy related 2B	Other	other
AT2A3_HUMAN	ATP2A3	ATPase, Ca++ transporting, ubiquitous	Cytoplasm	transporter
RENR_HUMAN	ATP6AP2	ATPase, H + transporting, lysosomal accessory protein 2	Cytoplasm	transporter
ATR_HUMAN	ATR	ataxia telangiectasia and Rad3 related	Nucleus	kinase
B4GT7_HUMAN	B4GALT7	xylosylprotein beta 1,4-galactosyltransferase, polypeptide 7	Cytoplasm	enzyme
BEND4_HUMAN	BEND4	BEN domain containing 4	Other	other
OSTCN_HUMAN	BGLAP	bone gamma-carboxyglutamate (gla) protein	Extracellular Space	other
BLM_HUMAN	BLM	Bloom syndrome, RecQ helicase-like	Nucleus	enzyme
BRCA2_HUMAN	BRCA2	breast cancer 2, early onset	Nucleus	transcription regulator
BRPF1_HUMAN	BRPF1	bromodomain and PHD finger containing, 1	Nucleus	transporter
CS068_HUMAN	C19orf68	chromosome 19 open reading frame 68	Other	other
CA174_HUMAN	C1orf174	chromosome 1 open reading frame 174	Nucleus	other
CA228_HUMAN	C1orf228	chromosome 1 open reading frame 228	Other	other
C1QC_HUMAN	C1QC	complement component 1, q subcomponent, C chain	Extracellular Space	other
CO3_HUMAN	C3	complement component 3	Extracellular Space	peptidase
CO4A_HUMAN	C4A/C4B	complement component 4B (Chido blood group)	Extracellular Space	other
C4BPA_HUMAN	C4BPA	complement component 4 binding protein, alpha	Extracellular Space	other
CI078_HUMAN	C9orf78	chromosome 9 open reading frame 78	Other	other
CAH3_HUMAN	CA3	carbonic anhydrase III, muscle specific	Cytoplasm	enzyme
CABIN_HUMAN	CABIN1	calcineurin binding protein 1	Nucleus	other
CAND1_HUMAN	CAND1	cullin-associated and neddylation-dissociated 1	Cytoplasm	transcription regulator
CAN1_HUMAN	CAPN1	calpain 1, (mu/I) large subunit	Cytoplasm	peptidase
CAN2_HUMAN	CAPN2	calpain 2, (m/II) large subunit	Cytoplasm	peptidase
CASC5_HUMAN	CASC5	cancer susceptibility candidate 5	Nucleus	other
C8AP2_HUMAN	CASP8AP2	caspase 8 associated protein 2	Nucleus	transcription regulator
CC154_HUMAN	CCDC154	coiled-coil domain containing 154	Other	other
CC171_HUMAN	CCDC171	coiled-coil domain containing 171	Other	other
CCD30_HUMAN	CCDC30	coiled-coil domain containing 30	Other	other
CCD37_HUMAN	CCDC37	coiled-coil domain containing 37	Other	other
CCD80_HUMAN	CCDC80	coiled-coil domain containing 80	Nucleus	other
CCHCR_HUMAN	CCHCR1	coiled-coil alpha-helical rod protein 1	Cytoplasm	other
CENPH_HUMAN	CENPH	centromere protein H	Nucleus	other
CP135_HUMAN	CEP135	centrosomal protein 135 kDa	Cytoplasm	other
CFAH_HUMAN	CFH	complement factor H	Extracellular Space	other
CHD4_HUMAN	CHD4	chromodomain helicase DNA binding protein 4	Nucleus	enzyme
CHD9_HUMAN	CHD9	chromodomain helicase DNA binding protein 9	Cytoplasm	other
ACHG_HUMAN	CHRNG	cholinergic receptor, nicotinic, gamma (muscle)	Plasma Membrane	transmembrane receptor
CHSTB_HUMAN	CHST11	carbohydrate (chondroitin 4) sulfotransferase 11	Cytoplasm	enzyme
CHSS3_HUMAN	CHSY3	chondroitin sulfate synthase 3	Cytoplasm	enzyme
CILP1_HUMAN	CILP	cartilage intermediate layer protein, nucleotide pyrophosphohydrolase	Extracellular Space	phosphatase
CLNK_HUMAN	CLNK	cytokine-dependent hematopoietic cell linker	Cytoplasm	other
CLUS_HUMAN	CLU	clusterin	Cytoplasm	other
CMBL_HUMAN	CMBL	carboxymethylenebutenolidase homolog (Pseudomonas)	Cytoplasm	enzyme
CNO6L_HUMAN	CNOT6L	CCR4-NOT transcription complex, subunit 6-like	Cytoplasm	enzyme
COPA1_HUMAN	COL25A1	collagen, type XXV, alpha 1	Cytoplasm	other
CROCC_HUMAN	CROCC	ciliary rootlet coiled-coil, rootletin	Plasma Membrane	other
CSRN1_HUMAN	CSRNP1	cysteine-serine-rich nuclear protein 1	Nucleus	transcription regulator
DIAC_HUMAN	CTBS	chitobiase, di-N-acetyl-	Cytoplasm	enzyme
CUL9_HUMAN	CUL9	cullin 9	Cytoplasm	other
CWC25_HUMAN	CWC25	CWC25 spliceosome-associated protein homolog (S. cerevisiae)	Other	other
CP1A2_HUMAN	CYP1A2	cytochrome P450, family 1, subfamily A, polypeptide 2	Cytoplasm	enzyme
CP51A_HUMAN	CYP51A1	cytochrome P450, family 51, subfamily A, polypeptide 1	Cytoplasm	enzyme
DAPL1_HUMAN	DAPL1	death associated protein-like 1	Other	other
DCAF6_HUMAN	DCAF6	DDB1 and CUL4 associated factor 6	Nucleus	transcription regulator
DCR1B_HUMAN	DCLRE1B	DNA cross-link repair 1B	Nucleus	enzyme
DCSTP_HUMAN	DCSTAMP	dendrocyte expressed seven transmembrane protein	Plasma Membrane	other
DCX_HUMAN	DCX	doublecortin	Cytoplasm	other
DDX51_HUMAN	DDX51	DEAD (Asp-Glu-Ala-Asp) box polypeptide 51	Other	enzyme
DEN2D_HUMAN	DENND2D	DENN/MADD domain containing 2D	Cytoplasm	other
DESM_HUMAN	DES	desmin	Cytoplasm	other
DGAT1_HUMAN	DGAT1	diacylglycerol O-acyltransferase 1	Cytoplasm	enzyme
DGC14_HUMAN	DGCR14	DiGeorge syndrome critical region gene 14	Nucleus	other
DHX30_HUMAN	DHX30	DEAH (Asp-Glu-Ala-His) box helicase 30	Nucleus	enzyme
DIP2B_HUMAN	DIP2B	DIP2 disco-interacting protein 2 homolog B (Drosophila)	Cytoplasm	other
DMXL1_HUMAN	DMXL1	Dmx-like 1	Extracellular Space	other
DYH17_HUMAN	DNAH17	dynein, axonemal, heavy chain 17	Cytoplasm	other
DYH2_HUMAN	DNAH2	dynein, axonemal, heavy chain 2	Other	other
DYH3_HUMAN	DNAH3	dynein, axonemal, heavy chain 3	Extracellular Space	enzyme
DYH5_HUMAN	DNAH5	dynein, axonemal, heavy chain 5	Cytoplasm	enzyme
DNJC7_HUMAN	DNAJC7	DnaJ (Hsp40) homolog, subfamily C, member 7	Cytoplasm	other
DOP1_HUMAN	DOPEY1	dopey family member 1	Cytoplasm	other
DSCAM_HUMAN	DSCAM	Down syndrome cell adhesion molecule	Plasma Membrane	other
DUS3L_HUMAN	DUS3L	dihydrouridine synthase 3-like (S. cerevisiae)	Other	other
DYHC2_HUMAN	DYNC2H1	dynein, cytoplasmic 2, heavy chain 1	Cytoplasm	other
COE2_HUMAN	EBF2	early B-cell factor 2	Nucleus	other
EBP_HUMAN	EBP	emopamil binding protein (sterol isomerase)	Cytoplasm	enzyme
EIF3C_HUMAN	EIF3C	eukaryotic translation initiation factor 3, subunit C	Other	translation regulator
ENPP1_HUMAN	ENPP1	ectonucleotide pyrophosphatase/phosphodiesterase 1	Plasma Membrane	enzyme
ENPP5_HUMAN	ENPP5	ectonucleotide pyrophosphatase/phosphodiesterase 5 (putative)	Extracellular Space	enzyme
PERE_HUMAN	EPX	eosinophil peroxidase	Cytoplasm	enzyme
EXOS1_HUMAN	EXOSC1	exosome component 1	Nucleus	enzyme
F150A_HUMAN	FAM150A	family with sequence similarity 150, member A	Other	other
F196B_HUMAN	FAM196B	family with sequence similarity 196, member B	Other	other
F208B_HUMAN	FAM208B	family with sequence similarity 208, member B	Other	other
YV021_HUMAN	FAM230B	family with sequence similarity 230, member B (non-protein coding)	Extracellular Space	other
FA78B_HUMAN	FAM78B	family with sequence similarity 78, member B	Other	other
FBF1_HUMAN	FBF1	Fas (TNFRSF6) binding factor 1	Nucleus	other
FIBA_HUMAN	FGA	fibrinogen alpha chain	Extracellular Space	other
FIBB_HUMAN	FGB	fibrinogen beta chain	Extracellular Space	other
FR1OP_HUMAN	FGFR1OP	FGFR1 oncogene partner	Cytoplasm	kinase
FGRL1_HUMAN	FGFRL1	fibroblast growth factor receptor-like 1	Plasma Membrane	transmembrane receptor
FIBG_HUMAN	FGG	fibrinogen gamma chain	Extracellular Space	other
FHAD1_HUMAN	FHAD1	forkhead-associated (FHA) phosphopeptide binding domain 1	Other	other
FIGL2_HUMAN	FIGNL2	fidgetin-like 2	Other	other
FLNB_HUMAN	FLNB	filamin B, beta	Cytoplasm	other
FINC_HUMAN	FN1	fibronectin 1	Extracellular Space	enzyme
FRMD3_HUMAN	FRMD3	FERM domain containing 3	Other	other
G6PC2_HUMAN	G6PC2	glucose-6-phosphatase, catalytic, 2	Cytoplasm	phosphatase
GAK_HUMAN	GAK	cyclin G associated kinase	Nucleus	kinase
GSH0_HUMAN	GCLM	glutamate-cysteine ligase, modifier subunit	Cytoplasm	enzyme
GCN1L_HUMAN	GCN1L1	GCN1 general control of amino-acid synthesis 1-like 1 (yeast)	Cytoplasm	translation regulator
CXB1_HUMAN	GJB1	gap junction protein, beta 1, 32 kDa	Plasma Membrane	transporter
GLRA2_HUMAN	GLRA2	glycine receptor, alpha 2	Plasma Membrane	ion channel
GMEB1_HUMAN	GMEB1	glucocorticoid modulatory element binding protein 1	Nucleus	transcription regulator
GOGA3_HUMAN	GOLGA3	golgin A3	Cytoplasm	transporter
AATC_HUMAN	GOT1	glutamic-oxaloacetic transaminase 1, soluble	Cytoplasm	enzyme
GRID2_HUMAN	GRID2	glutamate receptor, ionotropic, delta 2	Plasma Membrane	ion channel
GSAP_HUMAN	GSAP	gamma-secretase activating protein	Cytoplasm	peptidase
GSAS1_HUMAN	GSN-AS1	GSN antisense RNA 1	Other	other
GSHB_HUMAN	GSS	glutathione synthetase	Cytoplasm	enzyme
HERC1_HUMAN	HERC1	HECT and RLD domain containing E3 ubiquitin protein ligase family member 1	Cytoplasm	other
HES1_HUMAN	HES1	hes family bHLH transcription factor 1	Nucleus	transcription regulator
HILS1_HUMAN	HILS1	histone linker H1 domain, spermatid-specific 1, pseudogene	Nucleus	other
HIP1_HUMAN	HIP1	huntingtin interacting protein 1	Cytoplasm	other
HJURP_HUMAN	HJURP	Holliday junction recognition protein	Nucleus	other
HPTR_HUMAN	HPR	haptoglobin-related protein	Extracellular Space	peptidase
5HT2A_HUMAN	HTR2A	5-hydroxytryptamine (serotonin) receptor 2A, G protein-coupled	Plasma Membrane	G-protein coupled receptor
I23O2_HUMAN	IDO2	indoleamine 2,3-dioxygenase 2	Cytoplasm	enzyme
GILT_HUMAN	IFI30	interferon, gamma-inducible protein 30	Cytoplasm	enzyme
IGHA1_HUMAN	IGHA1	immunoglobulin heavy constant alpha 1	Extracellular Space	other
IGHG1_HUMAN	IGHG1	immunoglobulin heavy constant gamma 1 (G1m marker)	Extracellular Space	other
IGHM_HUMAN	IGHM	immunoglobulin heavy constant mu	Plasma Membrane	transmembrane receptor
IGJ_HUMAN	IGJ	immunoglobulin J polypeptide, linker protein for immunoglobulin alpha and mu polypeptides	Extracellular Space	other
IGKC_HUMAN	IGKC	immunoglobulin kappa constant	Extracellular Space	other
KV401_HUMAN	IGKV4-1	immunoglobulin kappa variable 4-1	Extracellular Space	other
LAC1_HUMAN	IGLC1	immunoglobulin lambda constant 1 (Mcg marker)	Cytoplasm	other
LAC2_HUMAN	IGLC2	immunoglobulin lambda constant 2 (Kern-Oz- marker)	Extracellular Space	other
IHH_HUMAN	IHH	indian hedgehog	Extracellular Space	enzyme
RED_HUMAN	IK	IK cytokine, down-regulator of HLA II	Extracellular Space	cytokine
IL1AP_HUMAN	IL1RAP	interleukin 1 receptor accessory protein	Plasma Membrane	transmembrane receptor
IRPL2_HUMAN	IL1RAPL2	interleukin 1 receptor accessory protein-like 2	Plasma Membrane	transmembrane receptor
IL26_HUMAN	IL26	interleukin 26	Extracellular Space	cytokine
INCE_HUMAN	INCENP	inner centromere protein antigens 135/155 kDa	Nucleus	other
IQCF6_HUMAN	IQCF6	IQ motif containing F6	Other	other
JARD2_HUMAN	JARID2	jumonji, AT rich interactive domain 2	Nucleus	transcription regulator
KTNB1_HUMAN	KATNB1	katanin p80 (WD repeat containing) subunit B 1	Cytoplasm	enzyme
KCND2_HUMAN	KCND2	potassium voltage-gated channel, Shal-related subfamily, member 2	Plasma Membrane	ion channel
KCNQ5_HUMAN	KCNQ5	potassium voltage-gated channel, KQT-like subfamily, member 5	Plasma Membrane	ion channel
KDM2B_HUMAN	KDM2B	lysine (K)-specific demethylase 2B	Nucleus	other
KDM5A_HUMAN	KDM5A	lysine (K)-specific demethylase 5A	Nucleus	transcription regulator
TALD3_HUMAN	KIAA0586	KIAA0586	Cytoplasm	other
K1161_HUMAN	KIAA1161	KIAA1161	Nucleus	other
KI13A_HUMAN	KIF13A	kinesin family member 13A	Cytoplasm	transporter
KIF19_HUMAN	KIF19	kinesin family member 19	Extracellular Space	enzyme
KIRR1_HUMAN	KIRREL	kin of IRRE like (Drosophila)	Plasma Membrane	other
KLC2_HUMAN	KLC2	kinesin light chain 2	Cytoplasm	other
KLRF1_HUMAN	KLRF1	killer cell lectin-like receptor subfamily F, member 1	Plasma Membrane	transmembrane receptor
LDB1_HUMAN	LDB1	LIM domain binding 1	Nucleus	transcription regulator
LHPL3_HUMAN	LHFPL3	lipoma HMGIC fusion partner-like 3	Other	other
LIPC_HUMAN	LIPC	lipase, hepatic	Extracellular Space	enzyme
YP023_HUMAN	LOC100128265	uncharacterized LOC100128265	Other	other
LRP1B_HUMAN	LRP1B	low density lipoprotein receptor-related protein 1B	Plasma Membrane	transmembrane receptor
LTBP2_HUMAN	LTBP2	latent transforming growth factor beta binding protein 2	Extracellular Space	other
LY75_HUMAN	LY75	lymphocyte antigen 75	Plasma Membrane	transmembrane receptor
MACD1_HUMAN	MACROD1	MACRO domain containing 1	Cytoplasm	enzyme
MANF_HUMAN	MANF	mesencephalic astrocyte-derived neurotrophic factor	Extracellular Space	other
MLP3A_HUMAN	MAP1LC3A	microtubule-associated protein 1 light chain 3 alpha	Cytoplasm	other
MAP4_HUMAN	MAP4	microtubule-associated protein 4	Cytoplasm	other
MA7D3_HUMAN	MAP7D3	MAP7 domain containing 3	Cytoplasm	other
MBD5_HUMAN	MBD5	methyl-CpG binding domain protein 5	Nucleus	other
MDN1_HUMAN	MDN1	MDN1, midasin homolog (yeast)	Nucleus	other
MEX3B_HUMAN	MEX3B	mex-3 RNA binding family member B	Other	kinase
MFNG_HUMAN	MFNG	MFNG O-fucosylpeptide 3-beta-N-acetylglucosaminyltransferase	Cytoplasm	enzyme
MKL1_HUMAN	MKL1	megakaryoblastic leukemia (translocation) 1	Nucleus	transcription regulator
MRE11_HUMAN	MRE11A	MRE11 meiotic recombination 11 homolog A (S. cerevisiae)	Nucleus	enzyme
RM32_HUMAN	MRPL32	mitochondrial ribosomal protein L32	Cytoplasm	translation regulator
MYBA_HUMAN	MYBL1	v-myb avian myeloblastosis viral oncogene homolog-like 1	Nucleus	transcription regulator
MYO15_HUMAN	MYO15A	myosin XVA	Cytoplasm	other
MYO3A_HUMAN	MYO3A	myosin IIIA	Cytoplasm	kinase
MYO6_HUMAN	MYO6	myosin VI	Cytoplasm	other
ULA1_HUMAN	NAE1	NEDD8 activating enzyme E1 subunit 1	Cytoplasm	enzyme
NUCL_HUMAN	NCL	nucleolin	Nucleus	other
NCOA2_HUMAN	NCOA2	nuclear receptor coactivator 2	Nucleus	transcription regulator
NEBU_HUMAN	NEB	nebulin	Cytoplasm	other
NEDD4_HUMAN	NEDD4	neural precursor cell expressed, developmentally down-regulated 4, E3 ubiquitin protein ligase	Cytoplasm	enzyme
NHS_HUMAN	NHS	Nance-Horan syndrome (congenital cataracts and dental anomalies)	Nucleus	other
NOA1_HUMAN	NOA1	nitric oxide associated 1	Cytoplasm	other
NRX3A_HUMAN	NRXN3	neurexin 3	Other	transporter
NSD1_HUMAN	NSD1	nuclear receptor binding SET domain protein 1	Nucleus	transcription regulator
NSN5C_HUMAN	NSUN5P2	NOP2/Sun domain family, member 5 pseudogene 2	Other	other
NET5_HUMAN	NTN5	netrin 5	Other	other
NUD15_HUMAN	NUDT15	nudix (nucleoside diphosphate linked moiety X)-type motif 15	Cytoplasm	phosphatase
OBSCN_HUMAN	OBSCN	obscurin, cytoskeletal calmodulin and titin-interacting RhoGEF	Cytoplasm	kinase
OCEL1_HUMAN	OCEL1	occludin/ELL domain containing 1	Other	other
ODFP2_HUMAN	ODF2	outer dense fiber of sperm tails 2	Cytoplasm	other
NOE2_HUMAN	OLFM2	olfactomedin 2	Cytoplasm	other
OPN4_HUMAN	OPN4	opsin 4	Plasma Membrane	G-protein coupled receptor
OR4K1_HUMAN	OR4K1	olfactory receptor, family 4, subfamily K, member 1	Plasma Membrane	G-protein coupled receptor
PALB2_HUMAN	PALB2	partner and localizer of BRCA2	Nucleus	other
PAR3L_HUMAN	PARD3B	par-3 family cell polarity regulator beta	Plasma Membrane	other
PARP4_HUMAN	PARP4	poly (ADP-ribose) polymerase family, member 4	Cytoplasm	enzyme
PCDH8_HUMAN	PCDH8	protocadherin 8	Plasma Membrane	other
PCLO_HUMAN	PCLO	piccolo presynaptic cytomatrix protein	Cytoplasm	transporter
PEAK1_HUMAN	PEAK1	pseudopodium-enriched atypical kinase 1	Plasma Membrane	kinase
PEG10_HUMAN	PEG10	paternally expressed 10	Nucleus	other
PER3_HUMAN	PER3	period circadian clock 3	Nucleus	other
PFD6_HUMAN	PFDN6	prefoldin subunit 6	Cytoplasm	other
PIGS_HUMAN	PIGS	phosphatidylinositol glycan anchor biosynthesis, class S	Cytoplasm	enzyme
P3C2A_HUMAN	PIK3C2A	phosphatidylinositol-4-phosphate 3-kinase, catalytic subunit type 2 alpha	Cytoplasm	kinase
SOX_HUMAN	PIPOX	pipecolic acid oxidase	Cytoplasm	enzyme
PLCD3_HUMAN	PLCD3	phospholipase C, delta 3	Cytoplasm	enzyme
PLXA4_HUMAN	PLXNA4	plexin A4	Plasma Membrane	transmembrane receptor
PNKD_HUMAN	PNKD	paroxysmal nonkinesigenic dyskinesia	Nucleus	other
PNKP_HUMAN	PNKP	polynucleotide kinase 3'-phosphatase	Nucleus	kinase
DPOLQ_HUMAN	POLQ	polymerase (DNA directed), theta	Nucleus	enzyme
PMGT1_HUMAN	POMGNT1	protein O-linked mannose N-acetylglucosaminyltransferase 1 (beta 1,2-)	Cytoplasm	enzyme
PPIG_HUMAN	PPIG	peptidylprolyl isomerase G (cyclophilin G)	Nucleus	enzyme
PP12C_HUMAN	PPP1R12C	protein phosphatase 1, regulatory subunit 12C	Cytoplasm	phosphatase
PPT2_HUMAN	PPT2	palmitoyl-protein thioesterase 2	Cytoplasm	enzyme
PREB_HUMAN	PREB	prolactin regulatory element binding	Nucleus	transcription regulator
PPCEL_HUMAN	PREPL	prolyl endopeptidase-like	Other	peptidase
PRG4_HUMAN	PRG4	proteoglycan 4	Extracellular Space	other
PRP31_HUMAN	PRPF31	pre-mRNA processing factor 31	Nucleus	other
PRC2A_HUMAN	PRRC2A	proline-rich coiled-coil 2A	Cytoplasm	other
PSB3_HUMAN	PSMB3	proteasome (prosome, macropain) subunit, beta type, 3	Cytoplasm	peptidase
PRS7_HUMAN	PSMC2	proteasome (prosome, macropain) 26S subunit, ATPase, 2	Nucleus	peptidase
PTPRM_HUMAN	PTPRM	protein tyrosine phosphatase, receptor type, M	Plasma Membrane	phosphatase
PTTG3_HUMAN	PTTG3P	pituitary tumor-transforming 3, pseudogene	Other	other
PZP_HUMAN	PZP	pregnancy-zone protein	Extracellular Space	other
RAB10_HUMAN	RAB10	RAB10, member RAS oncogene family	Cytoplasm	enzyme
RB3GP_HUMAN	RAB3GAP1	RAB3 GTPase activating protein subunit 1 (catalytic)	Cytoplasm	other
RAB6A_HUMAN	RAB6A	RAB6A, member RAS oncogene family	Cytoplasm	enzyme
RAB8B_HUMAN	RAB8B	RAB8B, member RAS oncogene family	Cytoplasm	enzyme
RGPA2_HUMAN	RALGAPA2	Ral GTPase activating protein, alpha subunit 2 (catalytic)	Cytoplasm	other
RBM23_HUMAN	RBM23	RNA binding motif protein 23	Nucleus	other
REG1A_HUMAN	REG1A	regenerating islet-derived 1 alpha	Extracellular Space	growth factor
RELN_HUMAN	RELN	reelin	Extracellular Space	peptidase
RFC4_HUMAN	RFC4	replication factor C (activator 1) 4, 37 kDa	Nucleus	other
RFX8_HUMAN	RFX8	RFX family member 8, lacking RFX DNA binding domain	Other	other
RMND1_HUMAN	RMND1	required for meiotic nuclear division 1 homolog (S. cerevisiae)	Cytoplasm	other
RNF17_HUMAN	RNF17	ring finger protein 17	Cytoplasm	other
RN213_HUMAN	RNF213	ring finger protein 213	Cytoplasm	enzyme
RN219_HUMAN	RNF219	ring finger protein 219	Other	other
FTM_HUMAN	RPGRIP1L	RPGRIP1-like	Cytoplasm	other
RL29_HUMAN	RPL29	ribosomal protein L29	Cytoplasm	other
RL37_HUMAN	RPL37	ribosomal protein L37	Cytoplasm	other
KS6A4_HUMAN	RPS6KA4	ribosomal protein S6 kinase, 90 kDa, polypeptide 4	Cytoplasm	kinase
RTKN_HUMAN	RTKN	rhotekin	Cytoplasm	other
RYR2_HUMAN	RYR2	ryanodine receptor 2 (cardiac)	Plasma Membrane	ion channel
SAMD8_HUMAN	SAMD8	sterile alpha motif domain containing 8	Cytoplasm	other
SASH1_HUMAN	SASH1	SAM and SH3 domain containing 1	Extracellular Space	other
UTER_HUMAN	SCGB1A1	secretoglobin, family 1A, member 1 (uteroglobin)	Extracellular Space	cytokine
SCUB3_HUMAN	SCUBE3	signal peptide, CUB domain, EGF-like 3	Plasma Membrane	other
SPB9_HUMAN	SERPINB9	serpin peptidase inhibitor, clade B (ovalbumin), member 9	Cytoplasm	other
SET1A_HUMAN	SETD1A	SET domain containing 1A	Nucleus	ion channel
SHAN1_HUMAN	SHANK1	SH3 and multiple ankyrin repeat domains 1	Cytoplasm	other
SHAN3_HUMAN	SHANK3	SH3 and multiple ankyrin repeat domains 3	Plasma Membrane	other
CTL1_HUMAN	SLC44A1	solute carrier family 44 (choline transporter), member 1	Plasma Membrane	transporter
SNTAN_HUMAN	SNTN	sentan, cilia apical structure protein	Other	other
SOLH1_HUMAN	SOHLH1	spermatogenesis and oogenesis specific basic helix-loop-helix 1	Cytoplasm	transcription regulator
SPAG7_HUMAN	SPAG7	sperm associated antigen 7	Nucleus	other
SPA2L_HUMAN	SPATA2L	spermatogenesis associated 2-like	Other	other
CYTSB_HUMAN	SPECC1	sperm antigen with calponin homology and coiled-coil domains 1	Nucleus	other
SPO11_HUMAN	SPO11	SPO11 meiotic protein covalently bound to DSB	Nucleus	enzyme
SPTN5_HUMAN	SPTBN5	spectrin, beta, non-erythrocytic 5	Plasma Membrane	other
SRGP2_HUMAN	SRGAP2	SLIT-ROBO Rho GTPase activating protein 2	Cytoplasm	other
SRG2C_HUMAN	SRGAP2C	SLIT-ROBO Rho GTPase activating protein 2C	Other	other
SIA7B_HUMAN	ST6GALNAC2	ST6 (alpha-N-acetyl-neuraminyl-2,3-beta-galactosyl-1,3)-N-acetylgalactosaminide alpha-2,6-sialyltransferase 2	Cytoplasm	enzyme
STXB1_HUMAN	STXBP1	syntaxin binding protein 1	Cytoplasm	transporter
SP20H_HUMAN	SUPT20H	suppressor of Ty 20 homolog (S. cerevisiae)	Nucleus	other
SPT6H_HUMAN	SUPT6H	suppressor of Ty 6 homolog (S. cerevisiae)	Nucleus	transcription regulator
SVEP1_HUMAN	SVEP1	sushi, von Willebrand factor type A, EGF and pentraxin domain containing 1	Cytoplasm	other
SYNJ1_HUMAN	SYNJ1	synaptojanin 1	Cytoplasm	phosphatase
TADA3_HUMAN	TADA3	transcriptional adaptor 3	Nucleus	transcription regulator
TBX20_HUMAN	TBX20	T-box 20	Nucleus	transcription regulator
TDRD1_HUMAN	TDRD1	tudor domain containing 1	Cytoplasm	other
TET1_HUMAN	TET1	tet methylcytosine dioxygenase 1	Nucleus	other
THMS1_HUMAN	THEMIS	thymocyte selection associated	Cytoplasm	other
TLK2_HUMAN	TLK2	tousled-like kinase 2	Cytoplasm	kinase
TM131_HUMAN	TMEM131	transmembrane protein 131	Extracellular Space	other
T132C_HUMAN	TMEM132C	transmembrane protein 132C	Other	other
T151A_HUMAN	TMEM151A	transmembrane protein 151A	Other	other
TM232_HUMAN	TMEM232	transmembrane protein 232	Other	other
TNFA_HUMAN	TNF	tumor necrosis factor	Extracellular Space	cytokine
TPD54_HUMAN	TPD52L2	tumor protein D52-like 2	Cytoplasm	other
TRML4_HUMAN	TREML4	triggering receptor expressed on myeloid cells-like 4	Other	other
TRI32_HUMAN	TRIM32	tripartite motif containing 32	Nucleus	transcription regulator
TRI65_HUMAN	TRIM65	tripartite motif containing 65	Other	other
TARA_HUMAN	TRIOBP	TRIO and F-actin binding protein	Nucleus	other
TRIPB_HUMAN	TRIP11	thyroid hormone receptor interactor 11	Cytoplasm	transcription regulator
TROAP_HUMAN	TROAP	trophinin associated protein	Cytoplasm	peptidase
TRPC5_HUMAN	TRPC5	transient receptor potential cation channel, subfamily C, member 5	Plasma Membrane	ion channel
TSG13_HUMAN	TSGA13	testis specific, 13	Other	other
TTC12_HUMAN	TTC12	tetratricopeptide repeat domain 12	Other	other
TITIN_HUMAN	TTN	titin	Cytoplasm	kinase
GCP6_HUMAN	TUBGCP6	tubulin, gamma complex associated protein 6	Cytoplasm	other
TRXR3_HUMAN	TXNRD3	thioredoxin reductase 3	Cytoplasm	enzyme
UBQLN_HUMAN	UBQLNL	ubiquilin-like	Other	other
UCKL1_HUMAN	UCKL1	uridine-cytidine kinase 1-like 1	Cytoplasm	kinase
UGDH_HUMAN	UGDH	UDP-glucose 6-dehydrogenase	Nucleus	enzyme
USP9X_HUMAN	USP9X	ubiquitin specific peptidase 9, X-linked	Plasma Membrane	peptidase
UTRO_HUMAN	UTRN	utrophin	Plasma Membrane	transmembrane receptor
VP13C_HUMAN	VPS13C	vacuolar protein sorting 13 homolog C (S. cerevisiae)	Cytoplasm	other
WAC_HUMAN	WAC	WW domain containing adaptor with coiled-coil	Nucleus	other
WDR1_HUMAN	WDR1	WD repeat domain 1	Extracellular Space	other
WDR35_HUMAN	WDR35	WD repeat domain 35	Cytoplasm	other
WDR43_HUMAN	WDR43	WD repeat domain 43	Nucleus	other
WFDC3_HUMAN	WFDC3	WAP four-disulfide core domain 3	Extracellular Space	other
YIPF1_HUMAN	YIPF1	Yip1 domain family, member 1	Cytoplasm	other
NIPA_HUMAN	ZC3HC1	zinc finger, C3HC-type containing 1	Nucleus	other
ZFHX4_HUMAN	ZFHX4	zinc finger homeobox 4	Extracellular Space	other
ZF64B_HUMAN	ZFP64	ZFP64 zinc finger protein	Nucleus	other
ZN132_HUMAN	ZNF132	zinc finger protein 132	Nucleus	other
ZNF14_HUMAN	ZNF14	zinc finger protein 14	Nucleus	transcription regulator
ZN215_HUMAN	ZNF215	zinc finger protein 215	Nucleus	transcription regulator
Z286B_HUMAN	ZNF286B	zinc finger protein 286B	Other	other
ZN345_HUMAN	ZNF345	zinc finger protein 345	Nucleus	transcription regulator
ZN532_HUMAN	ZNF532	zinc finger protein 532	Other	other
ZN561_HUMAN	ZNF561	zinc finger protein 561	Nucleus	other
ZN624_HUMAN	ZNF624	zinc finger protein 624	Nucleus	other
ZNF74_HUMAN	ZNF74	zinc finger protein 74	Nucleus	other

List of common exosomal proteins are presented as Protein ID, Symbol, Entrez Gene Name, Location and type. No significant differences were observed en exosomal protein content from fresh or frozen plasma (coefficient of variation < 5%) after different freeze thawing cycle from the same sample.

### Placenta-derived exosome increased during first trimester in normal pregnancy

Pooled exosome-containing fractions (*i.e.* fractions 4 to 7) were further characterised by determining the number of exosome (NEP) and exosomal PLAP concentration in the serial samples of maternal plasma obtained during first trimester of pregnancy (*i.e*. 6–12 weeks).

**Figure 3:**
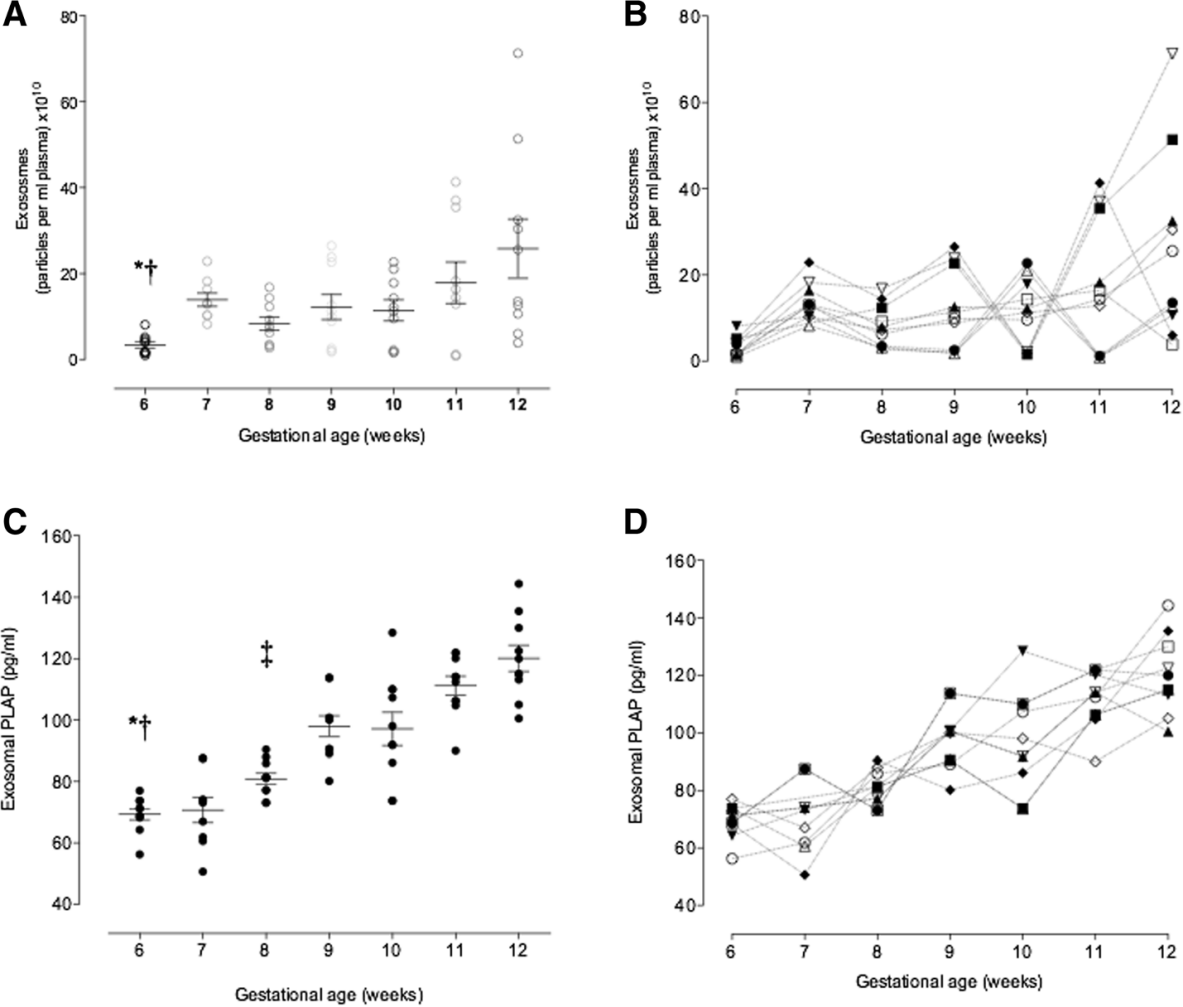
**Exosome profiling across first trimester pregnancy.** Enriched exosomal population (*i.e.* number of exosome particles) and placenta-derived exosomes (*i.e.* exosomal PLAP) were quantified in in peripheral plasma of women in the first trimester of pregnancy by ELISA. **(A)** exosomes as particles per ml plasma. **(B)** individual variation in exosome number for each week **(C)** exosomal PLAP during first trimester of pregnancy (*i.e.* 6–12 weeks). **(D)** individual variation in exosomal PLAP for each week. Data are presented as aligned dot plot and values are mean ± SEM. In **A**, two-way ANOVA ^**^p = 0.0048, Dunn’s post-hoc test analysis = ^*^p < 0.05 6 vs. 7 weeks and ^†^ *p* < 0.005: 6 vs. 12 weeks. In **C**, two-way ANOVA ^***^p < 0.0001, Dunn’s post-hoc test analysis = ^*^p < 0.05 6 vs. 9 and 10 weeks, ^†^ *p* < 0.005: 6 vs. 11 and 12 weeks, and ^‡^ *p* < 0.005: 8 vs. 11 and 12 weeks.

The gestational age variation in plasma exosome number was analysed by two-way ANOVA with the variance partitioned between gestational age and subject. A significantly effect of gestational age was identified (n = 69, one missing value, p < 0.005). A post-hoc multiple range test was used to identify statistically significant (p <0.05) differences between pairwise comparisons (Figure 3A). In addition, a significant effect of subject was identified (n = 69, one missing value, p < 0.05) (Figure 3B). In addition, NEP and gestational age (*i.e*. 6–12 weeks) displayed a significant positive linear relationship (r^2^ = 0.202, p < 0.001, n = 69, one missing value).

To assess gestational variation in placenta-derived exosomes, exosomal immunoreative (IR) PLAP was quantified using a commercial ELISA kit (see Methods). IR exosomal PLAP concentrations were analysed by two-way ANOVA with the variance partitioned between gestational age and subject. A significant effect of gestational age was identified (p < 0.0001, n = 69, one missing value) (Figure 3C). A post-hoc multiple range test was used to identify statistically significant (p <0.05) differences between pairwise comparisons (Figure 3D). No significant effect of patient on exosomal PLAP concentration was identified (p = 0.123). Immunoreactive exosomal PLAP concentration and gestational age displayed a significant positive linear linear relationship (r^2^ = 0.711, p < 0.001, n = 69, one missing value).

### Specific placental-derived exosomes

**Figure 4:**
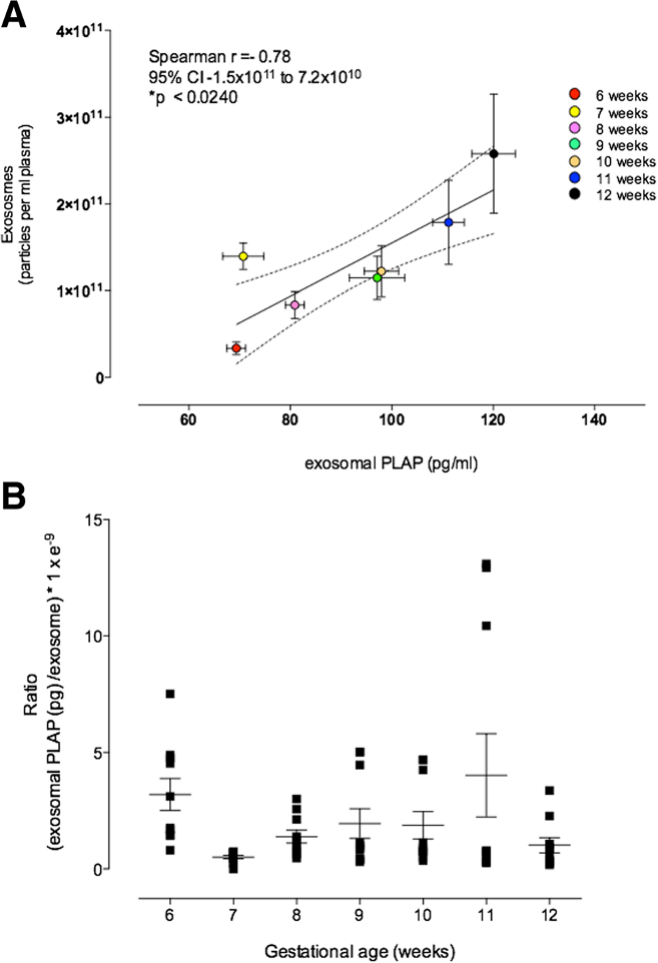
**Contribution of placental-derived exosomes into maternal circulation. (A)** Relationship between exosomal PLAP and exosomes (particles per ml plasma) across first trimester of pregnancy (i.e. 6–12 weeks represented by colours). **(B)** Ratio of specific placental exosome and exosomes. In **A**, values are mean ± SEM, Linear correlation (−). In **B**, Data are presented as aligned dot plot and values are mean ± SEM, two-way ANOVA p > 0.05.

Exosomal PLAP concentration and exosome number were subjected to linear regression analysis. The fitted linear model was described by the following equation: plasma exosomal PLAP pg/ml = 85.6 + 5.47 × 10^−11^ × exosome number/ml (p < 0.006, n = 69, one missing pair). The coefficient of determination (r^2^) was 10.8 (Figure 4A).

To estimate changes in the relative contribution of placental exosomes within the total exosomes present in maternal plasma and identify changes over the gestational age, the apparent PLAP content per 10^9^ exosome (PLAP ratio) was determined. Overall PLAP ratio averaged 2.01 ± 0.33 × 10^−9^ exosomal PLAP (pg) per exosome. The effects of gestational age on PLAP ratio were assessed by Kruskal-Wallis one-way ANOVA. No significant effect of gestational age on PLAP ratio was identified (*p* = 0.06) (Figure 4B).

## Discussion

Currently, there are no proven means of identifying presymptomatic women who subsequently develop complications of pregnancy during early pregnancy. Most women who are triaged into high-risk clinical units based on previous poor obstetric history ultimately have uncomplicated pregnancies. Available evidence supports the hypothesis that the aetiology of pregnancy complications begins during 1st trimester [15, 16]. If this is the case, profile of placenta-derived biomarkers during early pregnancy may be common between women with risk of developing pregnancy complications. Identification of such characteristics would provide opportunity to develop clinically useful early pregnancy screening tests.

Previously we have established that normal pregnancy is associated with the increase of exosomes into maternal plasma and the concentration of placenta-derived exosomes increases by 6-fold in uncomplicated healthy pregnancy during the first to third trimester [7] , however, the exosome profile in early pregnancy (*i.e.* from 6 to 12 weeks) remained to be established. The aim of this study was to characterise placenta-derived exosomes in maternal plasma over the first trimester of pregnancy and observe inter-subject variations in the exosome concentration. Weekly collected blood samples (from 6 to 12 weeks) were collected from normal healthy women to isolate and characterise the exosomes. The presence of exosomes were confirmed by: size (50–120 nM), and buoyant density (1.122- 1.197 g/ml). Endosomal (CD63) and placental (PLAP) antigens were identified in maternal plasma from as early as sixth week of pregnancy. The number of exosomes present in the maternal plasma increased progressively during the first trimester, as well as the exosomal PLAP concentration.

We isolated exosomes from the maternal plasma by differential and buoyant density centrifugation using a sucrose continuous gradient [7, 17]. The purification of exosomes from plasma and other biological fluids is not trivial, however, the use of an automatic system for fraction collection after the sucrose continuous gradient enable a high-reproducibility density, and decreasing the coefficient of variation between samples. In addition, using purification method based on the density of exosomes discards vesicles with the same size of exosomes with no endosomal origin, increasing the purity of exosome samples.

Previous studies have established that extracellular vesicles, including exosomes are released under physiological and pathophysiological conditions as well as during gestation [18]. The release of these vesicles is increased during pregnancy in response to different pathological conditions, presumably due to exosomal secretion from the placental trophoblast cells to the maternal peripheral circulation [19, 20] *.* In this study, we have established that exosomes are very stable when stored at −80°C. We obtained similar exosome yield from fresh and stored samples (*i.e.* plasma) and were able to identify gestational age differences in plasma exosome number in samples stored in long term. The isolation of exosomes from stored biofluids is the normal rather than the exception. These results are consistent with those of other studies [21, 22] suggesting that the exosomal content is protected inside these vesicles, highlighting the potential use of exosomes as biomarker for their high stability under different conditions.

As exosomes carry different kinds of protein, mRNA and miRNA [23], engaging in cell-to-cell communication, it is likely that they play an important role in modifying the maternal physiological state to maintain a successful pregnancy [24]. Interestingly, in this study we found that placental-derived exosomes increased systematically during the first trimester as early as sixth week of pregnancy when the intervillous circulation is not fully established. However, it has been observed that communication between placental and fetal circulation occurs at the beginning of the fourth week post conception [25]. Moreover, the lacunar spaces are formed in the trophoblast from as early as nine days post-ovulation and maternal blood flows into the trophoblast lacunae between ten and eleven days after fecundation. In addition, it has been reported that the intervillous blood flow is present in an early stage (*i.e.* < seventh week) [26] and increases gradually from fourth week during the first trimester of pregnancy [27].

Trophoblast plugs occlude the spiral arteries to prevent the contact of maternal blood flow into the intervillous space, however, at the same time trophoblast plug are in contact with the maternal blood, and could releases soluble proteins (*e.g.* human chorionic gonadotropin, hCG) and vesicles (*e.g*. nanovesicles) into maternal circulation. Interesting to highlight that hCG can be measured in maternal plasma as early as 4 weeks of gestation, confirming the presence of molecules released from the trophoblast in early pregnancy. Moreover, β-hCG and pregnancy-associated plasma protein A (PAPP-A) have been measured in maternal plasma as early as 6 weeks of gestation [28].

Specific placental-derived exosomes were quantified in the maternal circulation using the immunoreactive placental protein PLAP. Recent studies have demonstrated the presence of exosomes-PLAP^+ive^ only in peripheral circulation of pregnant women [7, 29]. PLAP is an integral membrane protein (enzyme) unique to the placenta (it has also been observed in some gynaecologic cancers), produced mainly by syncytiotrophoblast [30, 31]. Nevertheless, PLAP expression has been found in primary trophoblast cytotrophoblast cells [7] and ED27 trophoblast-like cells, both isolated from first trimester chorionic villi, and also in JEG-3 cells (a extravillous trophoblast model) [32]. In addition, using immunohistochemistry stain for PLAP, the majority of chorionic trophoblastic cells were positive for PLAP [33]. During the first trimester of pregnancy, the release of placental exosomes into the maternal blood may result from extravillous trophoblast and/or syncytiotrophoblast cells; however, while a definitive answer awaits further investigation, it is of relevance to note that fetal cells are present in maternal blood from 4 weeks of pregnancy and that trophoblast cells invade the decidua and myometrium from the time of implantation. Thus, a cellular and exosomal pathway exists for delivery into the maternal circulation.

Recently, several attempts and techniques were undertaken to determine and characterize the exosomal content in different biological fluids including normal human blood plasma [34–36]. As, the content of these released exosomes are placenta- specific [37], studying these nanovesicles is excellent method to understand the different processes occurring during embryo/fetal development and the feto-maternal interaction. Exosome analysis provides diagnostic and therapeutic potential, and biomarker opportunities for the early detection of diseases [38–40]. To date, several research studies have been performed to identify the morphologic and proteomic characteristics of exosomes released from the placental extravilous trophoblast cells and expression profile of these exosomal contents relates to common pregnancy conditions [8, 41, 42]. However, all these studies considered the late second or third trimester of pregnancy plasma samples for analysis.

## Conclusions

In conclusion, this study present longitudinal data on placental-derived exosomes in the first trimester of pregnancy, starting from as early as 6 weeks after implantation. Early detection of women at risk of complications of pregnancy would provide opportunity to evaluate appropriate intervention strategies to limit acute adverse squeal. The rationale for developing early pregnancy screening tests is not only for the management of the contemporaneous pregnancy but also to optimise lifelong and intergenerational health. If this can be achieved, it will provide an opportunity for early assignment of risk and the implementation of an alternative clinical management strategy to improve outcome for both the mother and baby.
